# Medical News

**Published:** 1851-04

**Authors:** 


### MEDICAL NEWS.

*Charleston*, 10*th March*, 1851

Editors of the Medical Examiner.

Gentlemen,—You will greatly oblige us by correcting, in your April issue, an error that crept into the notice of the time of meeting of the American Medical Association, contained in the January Number of the Charleston Medical Journal and Review. The *second* is, by mistake, put for the *first* Tuesday in May: the latter being the day prescribed by the Constitution for the meeting of that Board.

We are very respectfully your obedient servants,

D. J. Cain,
F. P. Porcher.

The Philadelphia Association for Medical Instruction, will commence its regular course of lectures for the summer, on Monday, April 7th. The general introductory will be delivered by Dr. West.

Graduates in Medicine, 1851.—Jefferson Medical College, 227. N. Y. University, 119. Maryland University, 45. Ohio Medical College, 59. University of Missouri, 83. Pennsylvania Medical College 36. Louisiana Medical College, 37.

Dr. Andrew Smith has been appointed to succeed Sir James McGrigor, as the head of the medical department of the British army.

Miss Martineau,—whose advocacy of homœopathy, mesmerism, and other kindred follies, has long been so notorious, now appears as the champion of the grossest and most undisguised materialism. In a late publication styled 'Letters on the laws of man's nature and development, by Henry George Atkinson F. G. S., and Harriet Martineau,' the lady's adoption of all the worn out trash of atheism is formally announced, as if to show the world how far the extremes of infidelity and credulity are compatible. An enthusiastic believer in homœopathy and the most ridiculous excesses of mesmerism, she entirely rejects the notion of a Great First Cause, a Providential Guidance, and, in short, of a spiritual life, and of all human responsibility and accountability. This is another proof, as has been well observed, that "the feebleness of mind which leads to the rejection of great truths, is precisely that which tends to the reception of as great errors."

POISONING BY MISTAKE.—One of those unfortunate errors which occasionally compromise, not only the reputation of the practitioner, but the life of the patient, has recently been the chief subject of conversation in the medical circles of Paris.

The head surgeon to one of the French lunatic asylums administered ten scruples instead of ten drops, of laudanum in lavement, to a patient. The patient died thirty-six hours afterwards, with doubtful symptoms. The error of prescription could not be denied, and the surgeon was therefore brought to trial.

Five of the most celebrated physicians in Paris declared, both as medical men and legal jurists, that it was not clear to them that death had arisen from poisoning with laudanum. On the other side, two professors at the *Veterinary* School of Alfort, unhesitatingly attributed the fatal effect to the narcotic poison. The surgeon was found guilty of involuncary homicide, and condemned to fifteen days' imprisonment. The jury, perhaps came to a correct conclusion; but it seems strange that their opinion should have been formed on evidence derived from the experience of men who deal exclusively in animals.

But opinions are formed in France on very slender grounds, and jurymen, if they go astray, may cite the example of ministers. In a public discussion the other day, at the National Assembly, M. Waisse, the Home Minister, in order to excuse the Government for its neglect of the poor, affirmed that of 21,000 children born in Manchester, only 300 attain the age of five years. In other words, the mortality of children in Manchester, up to the age of five years, is 98½ per cent. Surely this must be a grave and gross error. Even if the fact were true, it affords little consolation for the analogous one, "that in a certain part of Lille, only fonr children out of one hundred attain the age of five years."

The concours for the Chair of Surgery at the Faculty of Medicine still continues, and many of the exercises have been conducted in a brilliant manner. M. Robert, of the Hôtel-Dieu, and Professor Buisson, of Montpelier, appear to divide the chances between them. Either would make an excellent professor; Robert as a practical surgeon, Buisson as an accomplished teacher.—*Paris Correspondent of the London Med. Times* 22d. *Feb.* 1851.

Deaths of Distinguished Physicians.—The year 1850 has been fatal beyond all measure to medical science in France. Nearly all that remained to us illustrious in every department of the science has passed away. In chemistry Gay Lussac; in surgery, Marjorlin and Blandin; in comparative anatomy, de Blainville; in medicine, Fouquier, Royer-Collard, Capuron, Prus, and a host of others. To this long catalogue is to be added the name of M. Leuret, physician to the hospital of Bicêtre. M. Leuret was one of those persons, now so rare, who pursue their career without noise or ostentation; therefore, notwithstanding the extent and variety of his knowledge, the remarkable clearness of his judgment, and his personal value, it was long before M. Leuret enjoyed that degree of public estimation to which his peculiar talents so justly entitled him. He wanted ambition, and was consequently inactive. Even his great work on the Comparative Anatomy of the Nervous System still remains unfinished.

The peculiar doctrines of M. Leuret on the moral treatment of insanity are well known. They met with obstinate opposition in France, and were the principal cause of the little public success which their professor obtained. On the other hand, M. Leuret was one of the most effective opponents of phrenology, and his profound knowledge of the comparative anatomy of the brain enabled him to overthrow many a brilliant theory, which seemed inexpugnable when applied to man alone.

Naegele, the celebrated Professor of Midwifery of Hiedelberg, and Langenbech, the no less distinguished Professor of Anatomy and Surgery at Gottingen are dead. They died on the same day; Naegele at the age of 72, Langenbech at 75. Naegele was born at Dusseldorff and studied at Strasbourg and Paris. Langenbech, at his own expense, erected and endowed at Gottingen a Surgical Hospital and Anatomical Theatre.

Delegates from the Philadelphia County Medical Society.

*American Medical Association*,—Drs. B. H. Coates, G. Emerson, P. B. Goddard, Ed. Hallowell, B. S. Janney, J. F. Lamb, D. P. Lajus, Samuel Lewis, R. La Roche, J. H. B. McClellan, Lewis Rodman, Francis West, T. H. Yardley.

*State Medical Society*,—Drs. Wm. Ashmead, Henry Bond, T. F. Betton, D. F. Condie, G. Emerson, G. Fox, J. D. Griscom, I. Hays, N.

L. Hatfield, Prof. S. Jackson, Saml. Jackson (late of Northumberland), W. Jewell, J. F. Lamb, C. D. Meigs, W. Maybury, J. H. B. McClellan, G. W. Norris, Wm. B. Page, Isaac Parrish, Lewis Rodman, Isaac Remington, M. B. Smith, G. B. Wood, F. West, J. Warrington, T. H. Yardley.

